# Supplementary material for: Antioxidant Activity of Biogenic Cinnamic Acid Derivatives in Polypropylene
Source: Polymers (Basel). 2023 Sep 1;15(17):3621. doi: 10.3390/polym15173621 (PMC10490272; doi:10.3390/polym15173621)
Supplement: Supplementary file 1 [file polymers-15-03621-s001.zip › polymers-2512252-supplementary.pdf]

Antioxidant Activity of Biogenic Cinnamic Acid Derivatives in Polypropylene

Jannik Mayer [1], René Steinbrecher [2], Elke Metzsch-Zilligen [1] and Rudolf Pfaendner [1,*]

[1] Division Plastics, Fraunhofer Institute for Structural Durability and System Reliability LBF, Schlossgartenstraße 6, D-64289 Darmstadt, Germany
[2] Department Chemistry, University of Potsdam, Karl-Liebknecht-Str. 24-25, House 25, D-14476 Potsdam, Germany
* Correspondence: rudolf.pfaendner@lbf.fraunhofer.de

1. ^{1}H- and ^{13} C-NMR spectra of chemicals

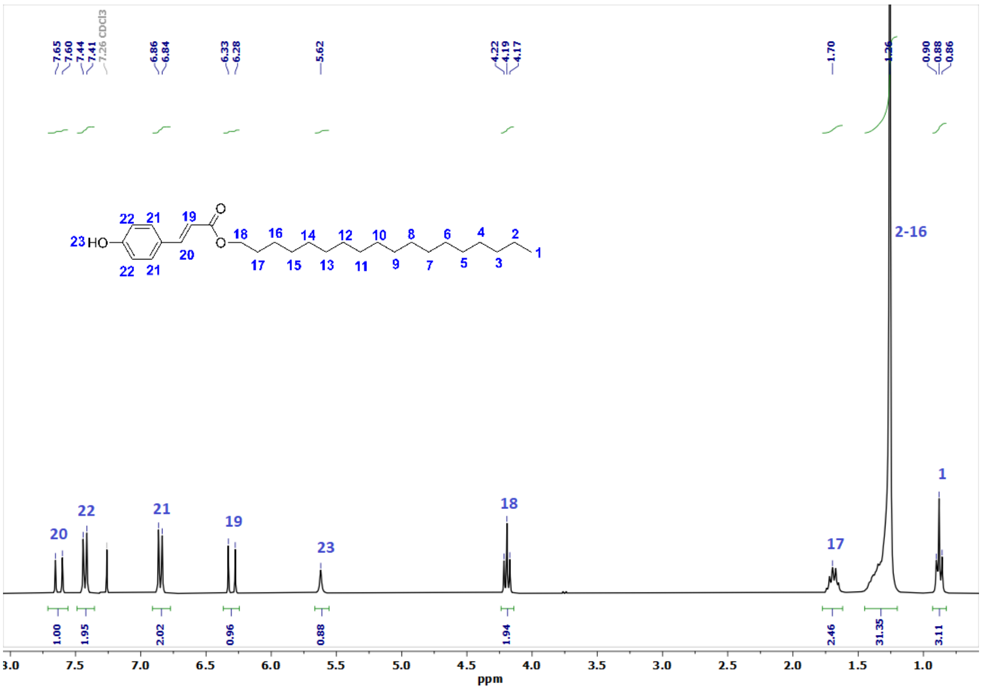

Figure S1 ^{1}H NMR of Hzocd in Chloroform-d.

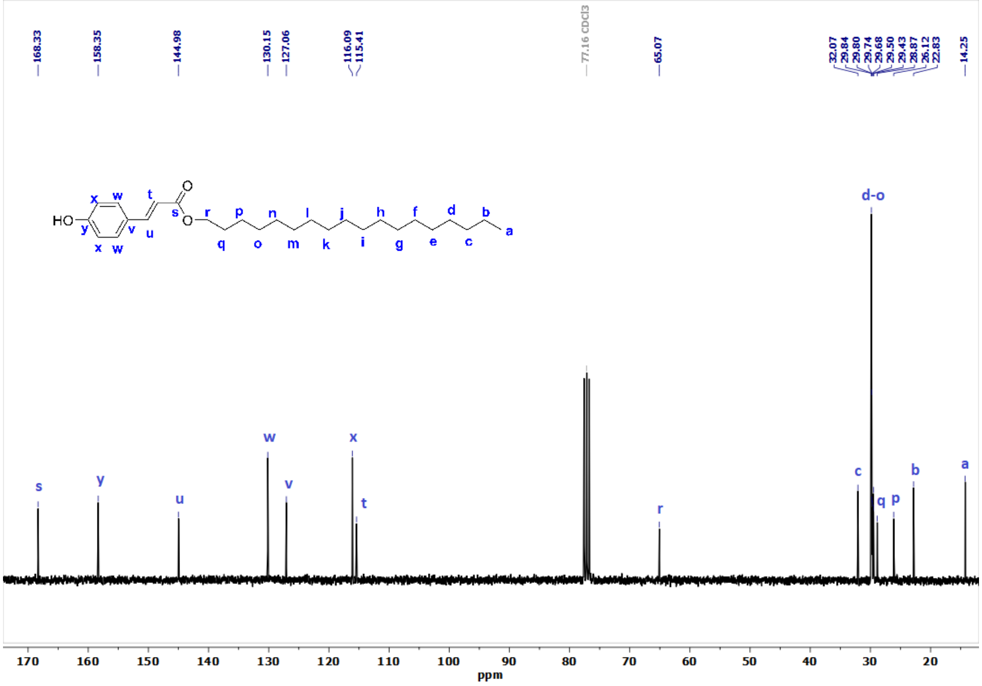

Figure S2 ^{13}C NMR of Hzocd in Chloroform-d.

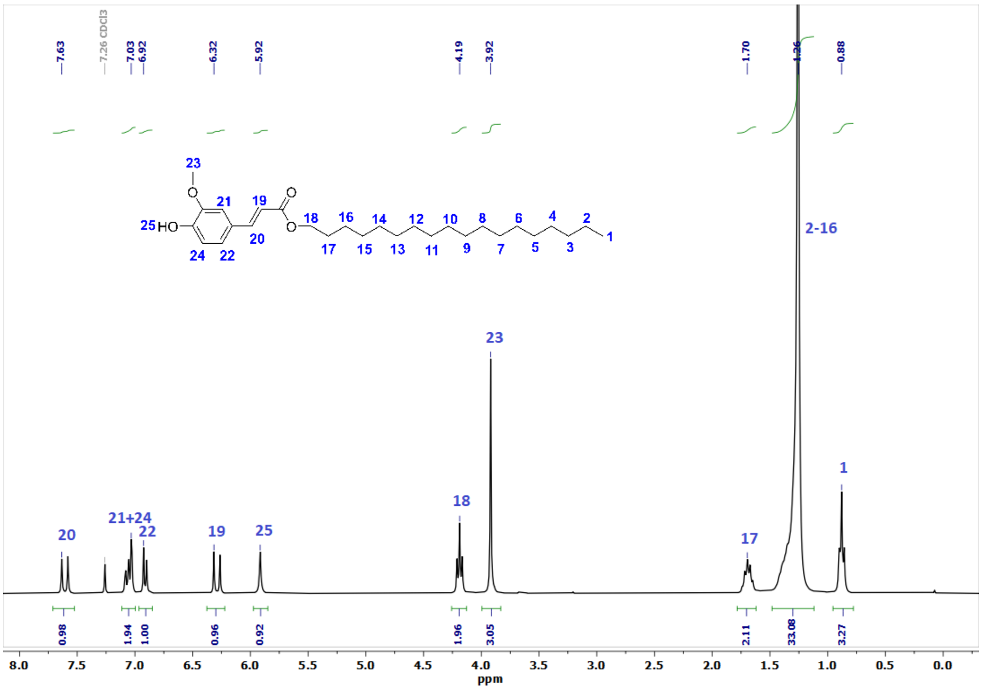

Figure S3 ^{1}H NMR of FaSa in Chloroform-d.

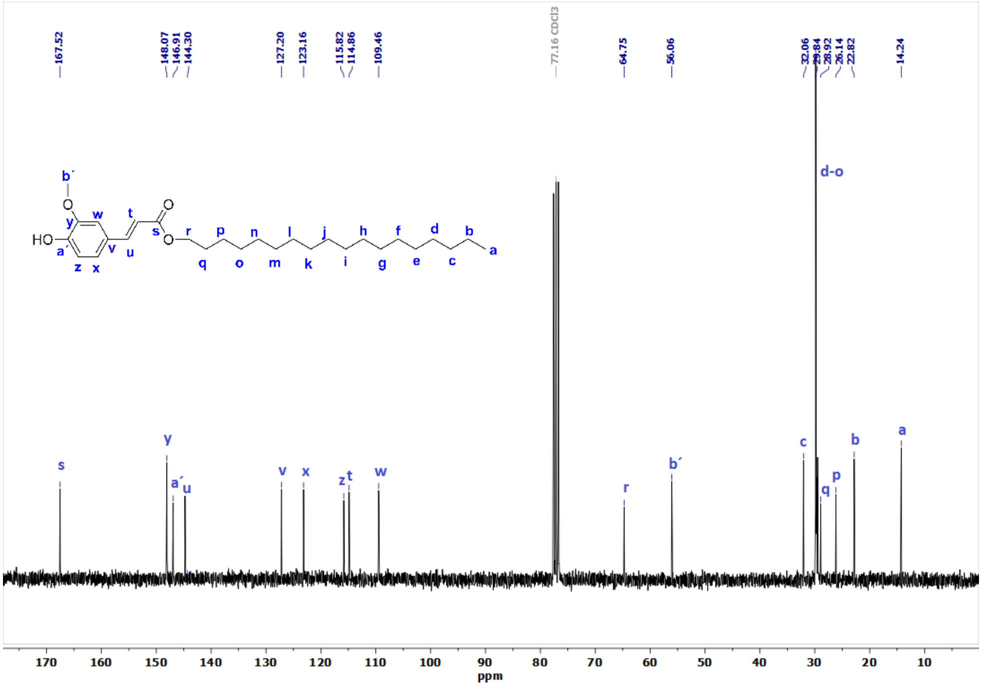

Figure S4 ^{13}C NMR of FaSa in Chloroform-d.

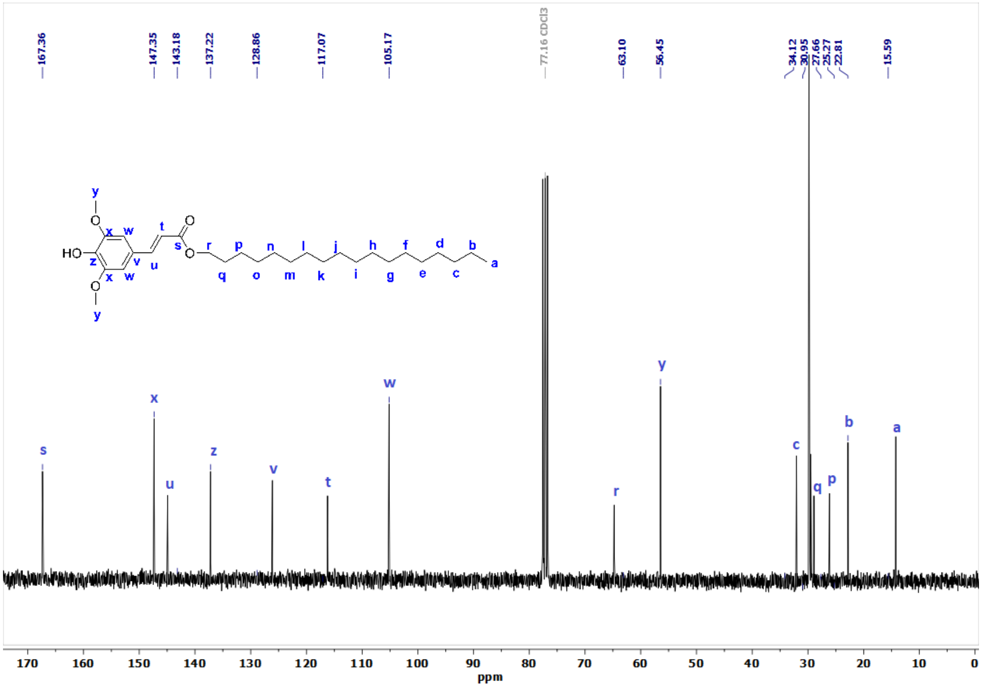

Figure S5 ^{13}C NMR of SinSa in Chloroform-d.

2. FTIR spectra

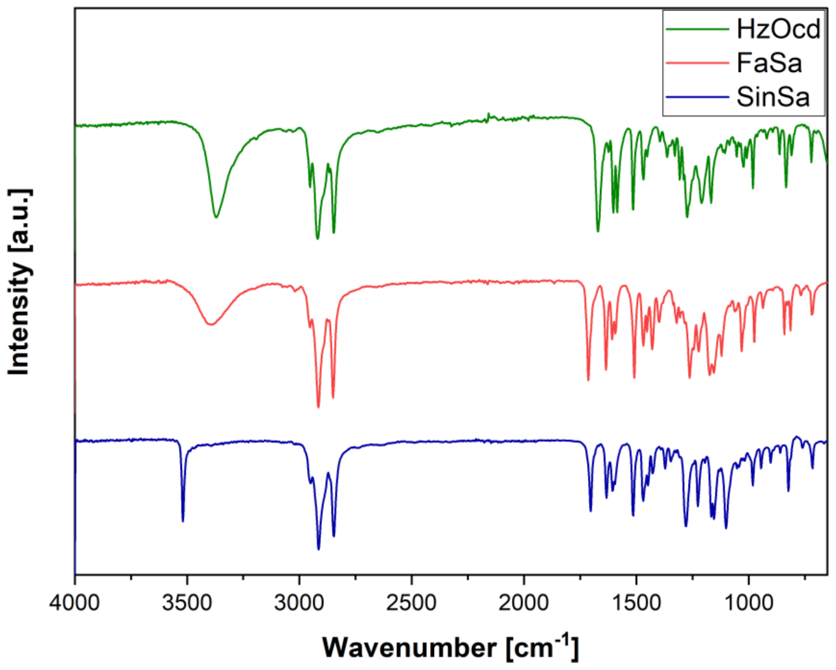

Figure S6 FTIR spectra of the synthesized hydroxycinnamic acid stearyl esters.

3. DPPH assay

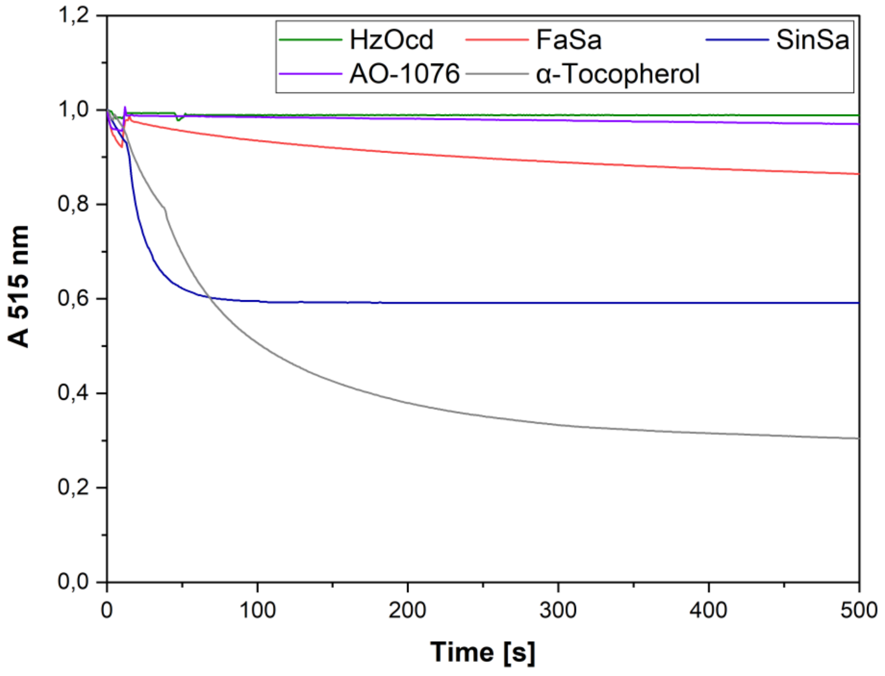

Figure S7 Decoloration kinetic curves of the examined antioxidants in the DPPH assay.

4. Performance as processing stabilizer

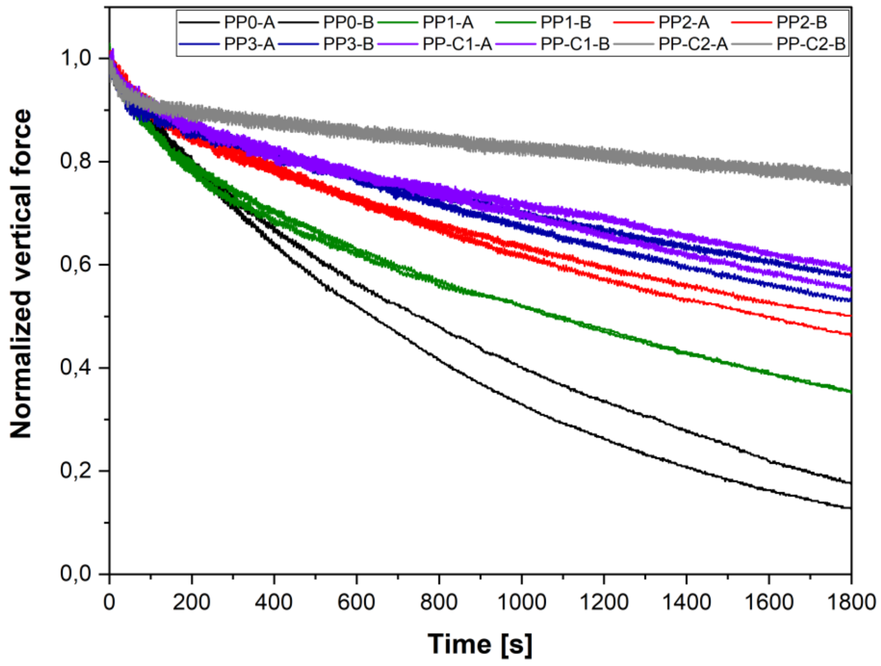

Figure S8 Obtained normalized vertical forces during 30 min micro-compounding with 200 °C and 200 rpm. Each compound is measured twice.

5. Long-term stability during accelerated aging

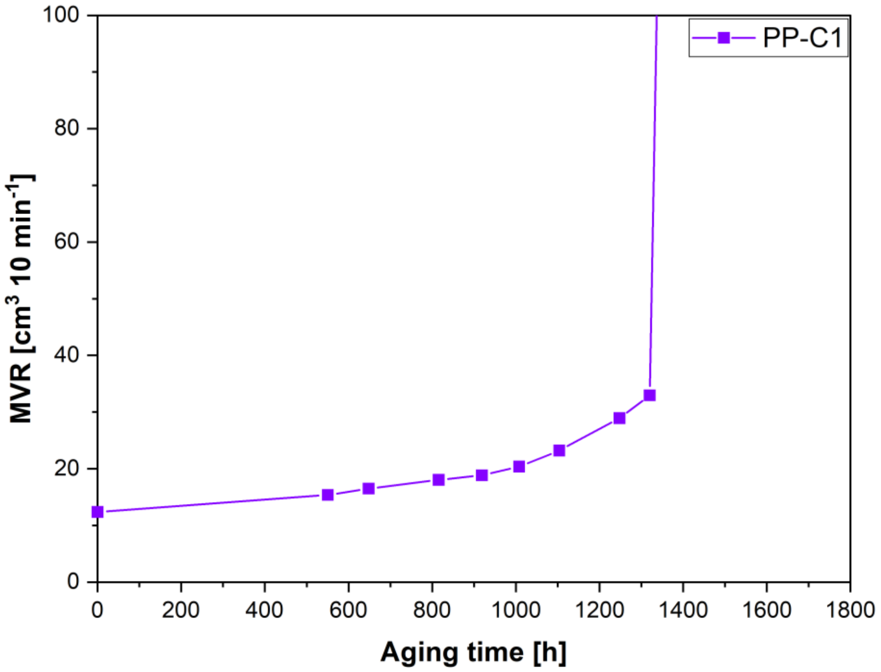

Figure S9 MVR values (2.16 kg/230 °C) during aging at 150 °C.

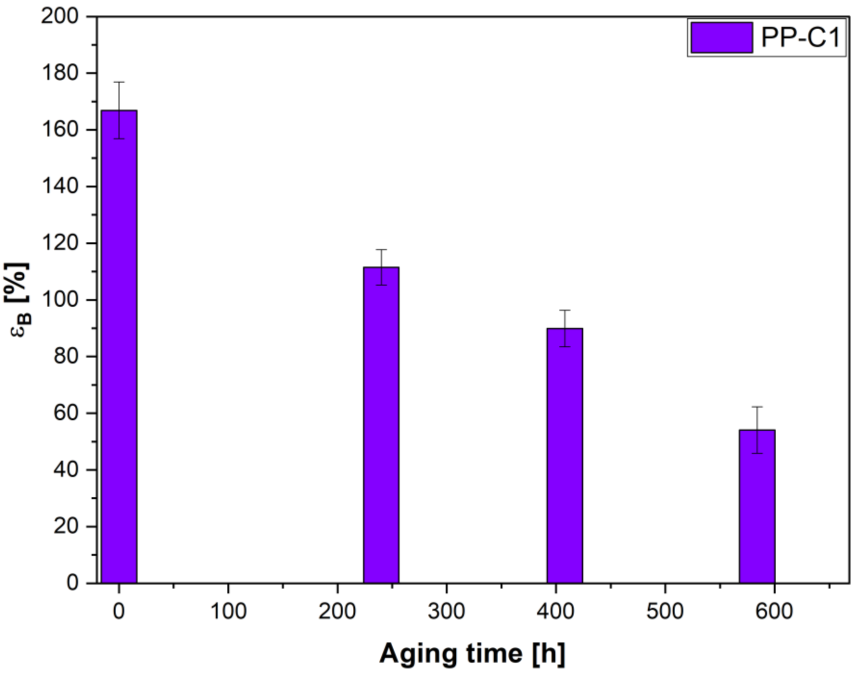

Figure S10 Elongation at break during aging at 150 °C.
